# Lateralized embodiment of ambiguous human silhouettes: Data on sex differences

**DOI:** 10.1016/j.dib.2019.104009

**Published:** 2019-05-23

**Authors:** Daniele Marzoli, Alessandra Pagliara, Giulia Prete, Gianluca Malatesta, Chiara Lucafò, Caterina Padulo, Alfredo Brancucci, Luca Tommasi

**Affiliations:** Department of Psychological, Health and Territorial Sciences, University of Chieti, Via dei Vestini 31, I-66100, Chieti, Italy

**Keywords:** Handedness, Human body, Ambiguous figures, Divided visual field paradigm, Lateralized embodiment, Sex differences

## Abstract

Whereas the role of observers' sex has already been addressed in research on embodied cognition, so far it has been neglected as regards laterality effects in embodied cognition. Here, we report further analyses of the data used in our paper “Hemispheric asymmetries in the processing of body sides: A study with ambiguous human silhouettes” [1], where participants had to indicate the perceived orientation of silhouettes with ambiguous front/back orientation and handedness presented in the right and left hemifield. Specifically, the variables examined in the associated paper (the number of right- and left-sided silhouettes perceived as front- and back-facing in each hemifield; the number of silhouettes perceived as right- and left-handed in each hemifield) are analyzed by also factoring in participant's sex). Moreover, data are provided and analyses are performed both for the total sample of participants and for the sample of right-handed participants only. For further details, as well as for the interpretation and discussion of the data, the reader is referred to the main article [1] and its Supplementary Material.

**Table:** Specifications Table

Subject area	*Neuroscience, Psychology*
More specific subject area	*Lateralization of embodied cognition*
Type of data	*Tables, figures*
How data was acquired	*Visual stimuli were presented, and the related data were recorded, using SuperLab 4.0 running on a Windows notebook with an Intel processor and a 15.4-inch monitor. Information on lateral preferences were collected with the Italian version of the Edinburgh Handedness Inventory**[2]*
Data format	*Calculated, analyzed*
Experimental factors	*One hundred and fifty-three participants (76 females and 77 males; 130 right-handers and 23 left-handers) were recruited. Fifteen participants were excluded because they reported awareness or suspicion about the experimental hypotheses or manipulations. The final sample consisted of 138 participants (74 females and 64 males; 116 right-handers and 22 left-handers)*
Experimental features	*Human silhouettes with ambiguous front/back orientation and handedness were presented in the right and left visual field on a notebook monitor. The task was to indicate the perceived orientation of the silhouettes. For each participant, two measures were computed: 1) the number of right- and left-sided silhouettes perceived as front- and back-facing in each hemifield, and 2) the number of silhouettes perceived as right- and left-handed in each hemifield*
Data source location	*Chieti, Italy*
Data accessibility	*Data are provided in this article*
Related research article	*Marzoli, D., Pagliara, A., Prete, G., Malatesta, G., Lucafò, C., Padulo, C., Brancucci, A., & Tommasi, L. Hemispheric asymmetries in the processing of body sides: A study with ambiguous human silhouettes. Neuroscience Letters, 656 (2017), 114–119*[1].

**Table:** 

**Value of the data** •The data and additional analyses made available here can provide useful information on sex differences in the domain of lateralized embodied cognition. •The data can be used for replication purposes, meta-analytic purposes, and exploratory analyses. •Data could be further statistically processed and analyzed. •The data can foster collaborations with other researchers and the design of further experiments in the area of embodied cognition.

## 1 Data

The excel files (Supplementary Files 1-8) accompanying this article contain a row for each participant, and the columns list several information including: personal data (sex, age, laterality score and handedness), whether the participant was excluded from analysis because of awareness about the experimental hypotheses or manipulations, the number of silhouettes perceived as right- and left-handed in each hemifield, the number of right- and left-sided silhouettes perceived as front- and back-facing in each hemifield, the number of catch trials perceived as front- and back-facing in each hemifield, and whether the participant was excluded from analysis because of being an outlier. Data are provided separately for the total sample of participants and for the sample of right-handed participants only. For both samples, four different statistical analyses are performed (one on the perceived orientation of the silhouettes factoring out participants' sex, one on the perceived handedness of the silhouettes factoring out participants' sex, one on the perceived orientation of the silhouettes factoring in participants' sex, and one on the perceived handedness of the silhouettes factoring in participants' sex), and outliers are computed accordingly. Statistics for significant effects are reported, and figures describing significant interactions are also included.

## 2 Experimental design, materials and methods

Data from 138 participants (74 females and 64 males; age: 19–52) were analyzed according to the exclusion criteria described in Marzoli, Pagliara, et al. [1]. The experimental design and methods are extensively described in the original article [1]. Thus, we briefly describe the paradigm and expand here only on the additional analyses that were not included therein.

We used the same stimuli as in Marzoli, Lucafò, et al. [3], consisting of 26 silhouettes of female and male persons performing one-handed manual actions printed in black against a white background, and their mirror images. Each original silhouette was mirrored horizontally in order to obtain a right-sided (from the observer's perspective) action (congruent with a right- or left-handed action if perceived as a back- or front-facing figure, respectively) and a left-sided action (congruent with a left- or right-handed action if perceived as a back- or front-facing figure, respectively). Moreover, 26 silhouettes of female and male persons who were not performing one-handed manual actions and the respective mirror images were used as catch trials. At a viewing distance of 57 cm, stimuli measured, on average, 6.8° horizontally and 10.7° vertically. The experiment was run using SuperLab 4.0 on a Windows notebook with an Intel processor and a 15.4-inch monitor. Participants were seated with their eyes about 57 cm from the computer screen. The experiment consisted of 208 trials (104 target trials and 104 catch trials) in which a black fixation cross presented for 500 ms in the center of a white screen was followed by a black silhouette presented laterally (9.1° from the fixation point) for 150 ms and then by a completely white screen. Participants were instructed to gaze at the fixation point and to indicate the perceived orientation of the stimulus. Each stimulus was presented twice, once to each hemifield. Finally, in order to assess the participant's hand preference, she/he was administered the Italian version of the Edinburgh Handedness Inventory [2]. Each participant was also probed for awareness of the purpose of the study.

For each participant, we computed: 1) the number of right- and left-sided silhouettes perceived as front- and back-facing in each hemifield, and 2) the number of silhouettes perceived as right- and left-handed in each hemifield. After removing outliers, these measures were used to perform eight separate repeated measures analyses of variance (ANOVAs). A first series of analyses aimed to test whether the perceived orientation of the figure was affected by the side in which the figure's action was represented and the hemifield of presentation. A second analysis aimed to test whether the perceived handedness of the figure was affected by the hemifield of presentation. Such analyses were performed both factoring out and factoring in participant's sex. Because of the low number of left-handed participants, it was not possible to include handedness (i.e., left or right manual dominance) as an independent variable, and thus laterality score (as measured by the Italian version of the Edinburgh Handedness Inventory) was included as a covariate in the analyses performed on the total sample. When needed, post-hoc two-tailed t-tests were carried out in order to specify the significant differences (p-values were adjusted using the Bonferroni correction for multiple comparisons within each set of post-hoc contrasts).

Outliers (i.e., participants who scored more than 2 standard deviations above or below the mean in any index) were computed either within the total sample of participants or within the sample of right-handed participants only, and either by collapsing sex or by separating female and male participants, according to whether data from left-handers were analyzed or not and according to whether sex was included as a factor or not. The eight ANOVAs performed are described below in Table 1, and the associated data and results are shown in the tables and figures of Supplementary Files (1-8).

**Table 1:** 

ANOVA	Sample	Within-subjects factors	Between-subjects factor	Covariate
First (see Supplementary File 1)	All participants	- Side of action	None	Laterality score
		- Perceived orientation of the figure		
		- Hemifield of presentation		
Second (see Supplementary File 2)	All participants	- Perceived handedness of the figure	None	Laterality score
		- Hemifield of presentation		
Third (see Supplementary File 3)	All participants	- Side of action	Participant's sex	Laterality score
		- Perceived orientation of the figure		
		- Hemifield of presentation		
Fourth (see Supplementary File 4)	All participants	- Perceived handedness of the figure	Participant's sex	Laterality score
		- Hemifield of presentation		
Fifth (see Supplementary File 5)	Right-handed participants	- Side of action	None	None
		- Perceived orientation of the figure		
		- Hemifield of presentation		
Sixth (see Supplementary File 6)	Right-handed participants	- Perceived handedness of the figure	None	None
		- Hemifield of presentation		
Seventh (see Supplementary File 7)	Right-handed participants	- Side of action	Participant's sex	None
		- Perceived orientation of the figure		
		- Hemifield of presentation		
Eighth (see Supplementary File 8)	Right-handed participants	- Perceived handedness of the figure	Participant's sex	None
		- Hemifield of presentation		
