# Supplementary material for: Lateralized embodiment of ambiguous human silhouettes: Data on sex differences
Source: Data Brief. 2019 May 23;25:104009. doi: 10.1016/j.dib.2019.104009 (PMC6545413; doi:10.1016/j.dib.2019.104009)
Supplement: Multimedia component 1 [file mmc1.docx]

*Data article*

**Lateralized embodiment of ambiguous human silhouettes: Data on sex differences**

**Daniele Marzoli^1*^, Alessandra Pagliara^1^, Giulia Prete^1^, Gianluca Malatesta^1^, Chiara Lucafò^1^, Caterina Padulo^1^, Alfredo Brancucci^1^, Luca Tommasi^1^**

*^1^Department of Psychological, Health and Territorial Sciences, University of Chieti, Via dei Vestini 31, I-66100, Chieti, Italy*

d.marzoli@unich.it

**Declarations of interest**: none.
